# Molecular dynamics simulations of nanoindentation and scratch in Cu grain boundaries

**DOI:** 10.3762/bjnano.8.228

**Published:** 2017-11-01

**Authors:** Shih-Wei Liang, Ren-Zheng Qiu, Te-Hua Fang

**Affiliations:** 1 Department of Mechanical Engineering, National Kaohsiung University of Applied Sciences, Kaohsiung 807, Taiwan

**Keywords:** indentation, molecular dynamics, nanograin boundary, nanoscratch

## Abstract

The dynamic nanomechanical characteristics of Cu films with different grain boundaries under nanoindentation and scratch conditions were studied by molecular dynamics (MD) simulations. The type of grain boundary is the main factor in the control of the substrate atoms with respect to the size of dislocations since the existence of the grain boundary itself restricts the movement associated with dislocations. In this work, we analyzed the transverse and vertical grain boundaries for different angles. From the simulation results, it was found that the sample with a transverse grain boundary angle of 20° had a higher barrier effect on the slip band as compared to samples with other angles. Moreover, the nanoindentation results (i.e., indentation on the upper area) of the vertical grain boundary showed that the force was translated along the grain boundary, thereby producing intergranular fractures.

## Introduction

The recent developments in nanotechnology have allowed the related research techniques to be applied to diverse fields including physics, energy, medicine, and industrial engineering, among others [1–2]. In this sense, nanoindentation [3–12] and nanoscratch [13–21] techniques have been used to measure the mechanical properties and wear resistance of materials with the aim to design nanoscale devices. On a microscopic level, the dislocation phenomenon of the internal material affects its structure. However, the observation of this slight change in the internal structure of the material along with the complex dislocation phenomenon of the grain boundary is unclear at the macroscopic scale. In the past few years, many researchers have used numerical simulation analysis to discuss the mechanical properties of metals [3–21].

Molecular dynamics (MD) simulation is an effective tool for studying material behavior and system design at the nanometer scale while avoiding experimental noise and turbulence issues. Landman et al. [7] studied the interactional properties (i.e., the characteristics of deformation and adhesion when a probe touches the substrate) between Ni (probe) and Au (thin film substrate) under nanoindentation conditions using MD simulations. Moreover, Mulliah et al. [18] analyzed the friction coefficient, the dislocation, and related parameters of the nanostructure under nanoscratch conditions using MD simulations. However, the nanomechanical properties of the different types of grain boundaries have been scarcely studied using MD simulations. Therefore, the study of the potential fracture of substrates upon mechanical pressure as a result of the different grain boundaries is of interest.

In this paper, the nanomechanical and grain boundary characteristics of Cu films were studied using MD simulations. The results are discussed in terms of the atomic trajectories, slip vectors, atomic flows, as well as the force and the average friction coefficients.

## Methodology

**Figure 1:**
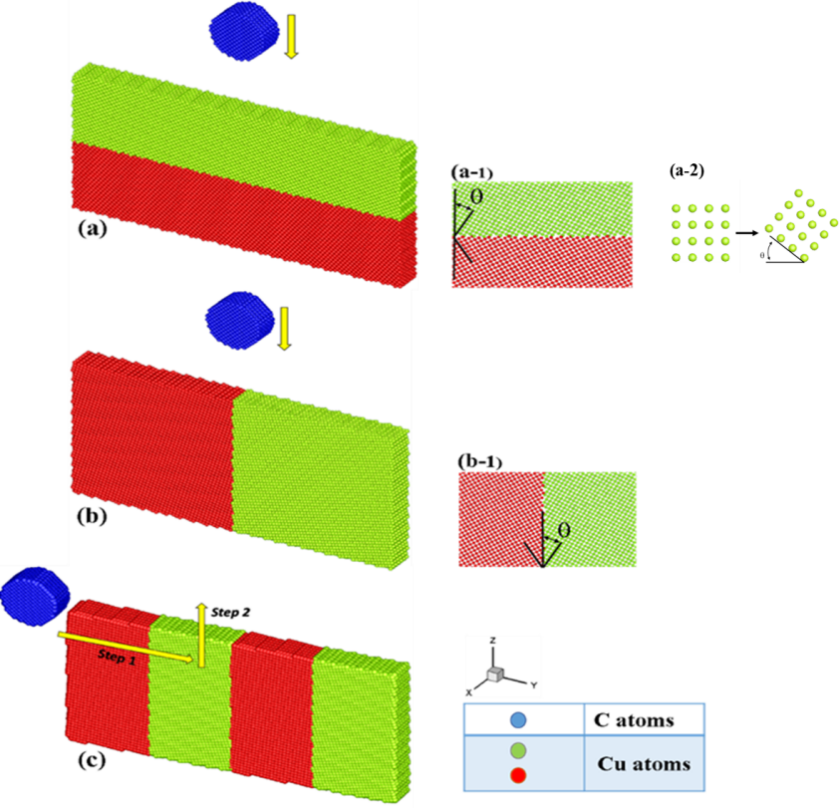
Physical model diagrams of (a) transverse grain boundary indentation, (b) vertical grain boundary indentation, (c) vertical grain boundary scratches, where subplots (a-1), (a-2) and (b-1) show how the Cu atoms of the substrate rotate around the *x*-axis with the angle ±θ.

The physical model diagrams used herein for analyzing the grain boundary properties (i.e., transverse grain boundary indentation, vertical grain boundary indentation, and vertical grain boundary scratches) are shown in Figure 1a–c, respectively. The indenter (blue) was made of a perfectly structured diamond while perfect face-centered cubic (FCC) Cu atoms comprised the substrate (red and green). With the aim to exclusively study the behavior of the substrate, the indenter was considered as a rigid body (i.e., the indenter wear was not investigated). However, the substrate atoms were set to follow a Newtonian behavior except the three layers of atoms at the bottom, which were fixed to support the entire system. The substrate (length, width, and height of 25, 2, and 12 nm, respectively) and the indenter (radius: 2 nm) were comprised of 55,162 and 4,538 atoms, respectively. The temperature of the system was set at 300 K. In this study, the substrate first undergoes a balance process for about 200 ps. The indentation and scratch processes will not begin until the substrate is stable. In the transverse grain boundary and multilayers, a periodic boundary condition was imposed in the *x* and *y* directions, while the *z* direction was considered to be the real size. Regarding the scratch for the vertical grain boundary, the *y* and *z* directions were considered to be the real size. The parameter was set according to the arrangement property for the *x*-axis, thereby preventing the boundary from affecting the simulation results. On the other hand, the edge stresses along the *y*-axis had a very small impact on simulation results since they were quite far from indenter, and can thus be neglected. Moreover, the Cu atoms of the substrate were represented by the colors red and green, depending on the rotation angle (±θ, where θ = 10–40°) around the *x*-axis, which was considered a special angle of the grain boundary (subplots Figure 1a-1 and Figure 1b-1. Moreover, the ensemble of the system was NVT, where T, V, and N, represent the temperature, the volume, and the number of the atoms, respectively, are fixed.

In the case of the transverse and vertical grain boundary indentation systems shown in Figure 1a,b, the indenter first pressed down on the *z*-axis at a velocity of 20 m/s and was held at these conditions for a period of time. Subsequently, the indenter was unloaded quickly at a speed twice that of the press. Moreover, in the case of the vertical grain boundary scratch systems (Figure 1c), the indenter was moved from the left to the right side of the substrate along the *y*-axis at a depth of 1 nm. The length and the speed of the scratch were 12 nm and 100 m/s, respectively.

The tight-binding second momentum approach (TB-SMA) [22] was used to describe the Cu–Cu interaction. The TB-SMA is expressed as

[1]
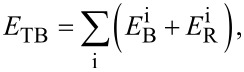


where 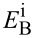
and 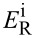
represent the repulsive and attractive potentials of atom i, respectively, which can be expressed as

[2]
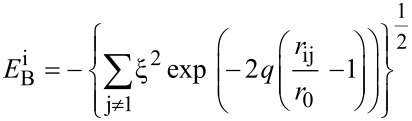


[3]
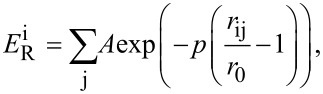


where *r*_ij_ is the distance between atoms i and j, while *r*_0_ is the first-neighbor distance. *A*, ξ, *p*, and *q* are adjustable parameters governing the interaction among atoms which were determined from the experimental cohesive energy, lattice parameter, bulk modulus, and elastic constant values, respectively. The parameters for the Cu–Cu interaction were *r*_0_ = 2.5 Å, ξ = 1.224 eV, *A* = 0.0855 eV, *p* = 10.96, and *q* = 2.278 [23]. Moreover, the Lennard–Jones (LJ) potential [24] was used to describe the interaction between the indenter and the substrate. The parameters of the LJ potential used in this study are based on the previous literature [25–29], which was expressed as Φ(γ) = 4ε[(σ/*r*)^12^ − (σ/*r*)^6^]. The parameters *r*, ε, and σ represent the distance between atoms, the binding energy, and the balance distance, respectively. The parameters ε and σ for the C–Cu interaction were selected as 0.05477 eV and 2.168 Å, respectively [23]. The cutoff distances for tight-binding (TB) and LJ potentials were 8.5 and 2.8 Å, respectively.

With the aim to observe the effect of the indentation and scratch grain boundaries on the atomic movement during dislocation, the diagram of the slip vector [30], which was applied to body-centered cubic (BCC), FCC, and hexagonal close packed (HCP) structures, was defined as

[4]
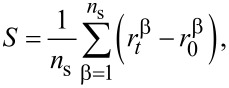


where *n*_s_ is the number of slipped neighbors and β represents the slipped neighbors. 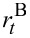 and 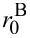 are the relative vectors between a given atom and its neighbor β at the current and initial times, respectively.

With the aim to further study the stress distribution of the substrate under nanoindentation and scratch processes, the atomic stress, σ_i_, was defined as

[5]
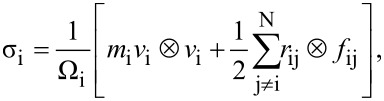


which is also called the Basinski–Duesbery–Taylor (BDT) stress. Ω_i_ is the effective atomic volume for atom i, *m*_i_ is the mass of atom i, *v*_i_ is the velocity of atom i, and *f*_ij_ and *r*_ij_ are the interatomic force and the distance between atoms i and j, respectively. The symbol 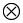 denotes the tensor product of two vectors. For the equivalent stress (i.e., the von Mises stress) [4], the BDT stress components are defined as

[6]
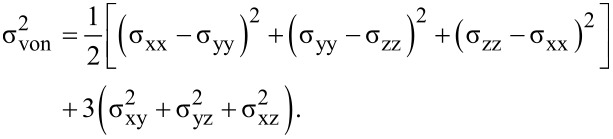


## Results and Discussion

### Indentation for the transverse grain boundary

**Figure 2:**
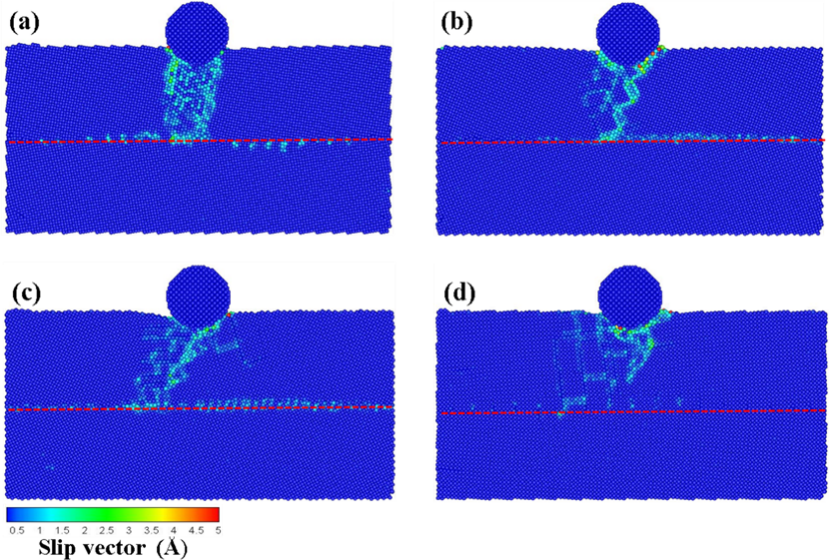
The slip vector diagrams of the transverse grain boundary with the different angles of (a) θ = 10°, (b) θ = 20°, (c) θ = 30°, and (d) θ = 40° for an indentation of 1 nm. The red dashed line corresponds to the grain boundary.

Figure 2a–d shows the slip vector diagrams of the transverse grain boundary at different angles (10–40°) for an indentation of 1 nm. As can be seen from Figure 2a, the slip area is found concentrated on the area under the indenter for the grain boundary with a 10° angle. In the cases of the 20° and 30° angle materials (Figure 2b,c, respectively) the slip areas showed a tendency to move to the left side. However, in the case of the grain boundary with a 40° angle, the slip areas were more evenly distributed around the indenter. These different results were produced by the different structures of the grain boundary.

**Figure 3:**
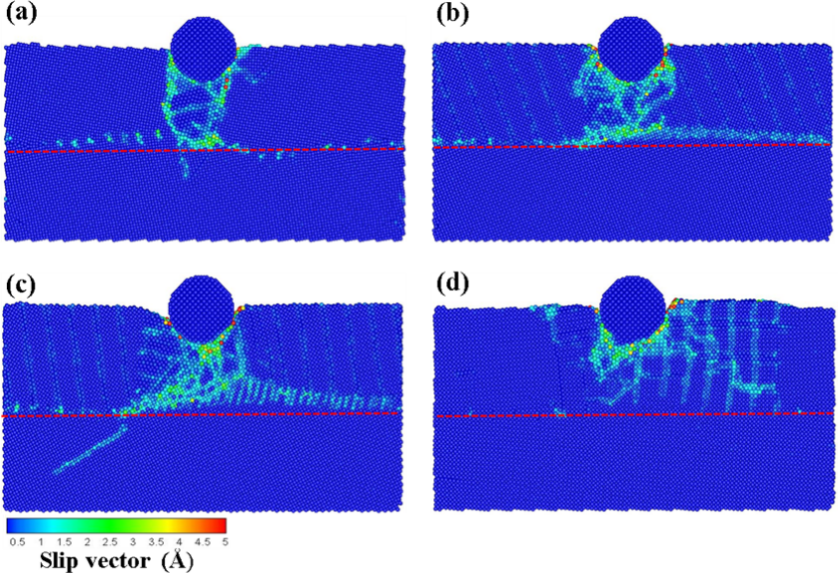
The slip vector diagrams of the transverse grain boundary with the different angles of (a) θ = 10°, (b) θ = 20°, (c) θ = 30°, and (d) θ = 40° for an indentation of 2 nm. The red dashed line corresponds to the grain boundary.

Next, the depth on the indenter was increased to 2 nm, and the results are shown in Figure 3a–d. In the case of the grain boundary with 10° and 30° angles (Figure 3a and c, respectively) the indentation force passed through the grain boundary and was transmitted to the lower substrate, which produced the dislocation. Additionally, the grain boundary with 10° angle was found to bend owing to the force of the press. Moreover, as shown in Figure 3b, a large number of dislocation pile-ups were found on the grain boundary. This behavior can be explained by a faster decay of the structural strength of the grain boundary as compared to the structure of the grain itself, thereby directing the destruction along the grain boundary rather than through the grain. This destruction phenomenon is denoted as intergranular fracture [20–21]. Cleri et al. [20] reported that the grain boundary in FCC Cu generates an asymmetric crack growth based on the MD simulation of an intergranular fracture. The authors reported that a crack propagated along the interface plane of the symmetric tilt grain boundary. No noticeable slip phenomenon on the grain boundary was found for the grain boundary with 40° angle (Figure 3d).

**Figure 4:**
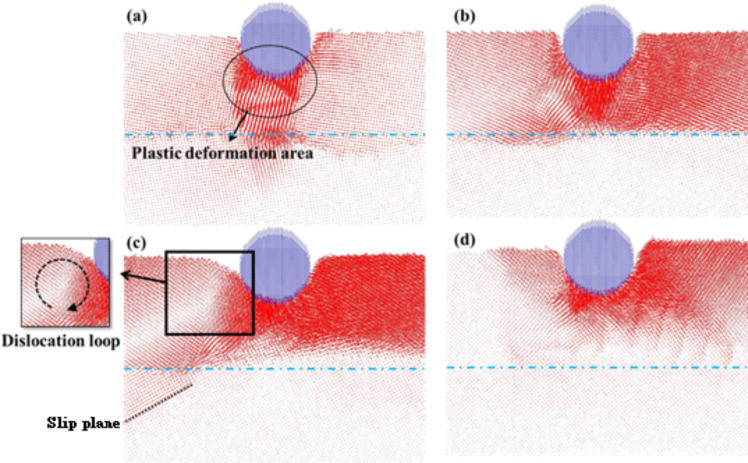
The atomic flow diagrams of the transverse grain boundary with the different angles of (a) θ = 10°, (b) θ = 20°, (c) θ = 30°, and (d) θ = 40° for an indentation of 2 nm. The blue dashed line corresponds to the grain boundary. The obvious grain boundary phenomena are marked.

Figure 4a–d shows the atomic flow diagrams of the transverse grain boundaries with different angles (θ = 10–40°, respectively) for an indentation of 2 nm. As shown in Figure 4a, the compressed atoms under the probe gave rise to the tight block of plastic deformation. Since the atoms in the upper layer of the grain boundary are blocked by the grain boundary, they moved to the left and right rather than to the lower layer of the grain boundary, thereby leading to an irregular, directional slip for the lower atoms (Figure 4b). As shown in Figure 4c, a dislocation loop appeared near the left side of the indenter as a result of the compression process. When the dislocation path through the grain boundary with the higher solidity is difficult, it starts to bend and shape around the slip plane, which induces atoms to continuously move. However, in the case of the 40° angle grain boundary (shown in Figure 4d), the atoms were displaced around the probe upon compression with no obvious dislocation accumulation at the grain boundary.

### Indentation for the transverse grain boundary with multilayers

**Figure 5:**
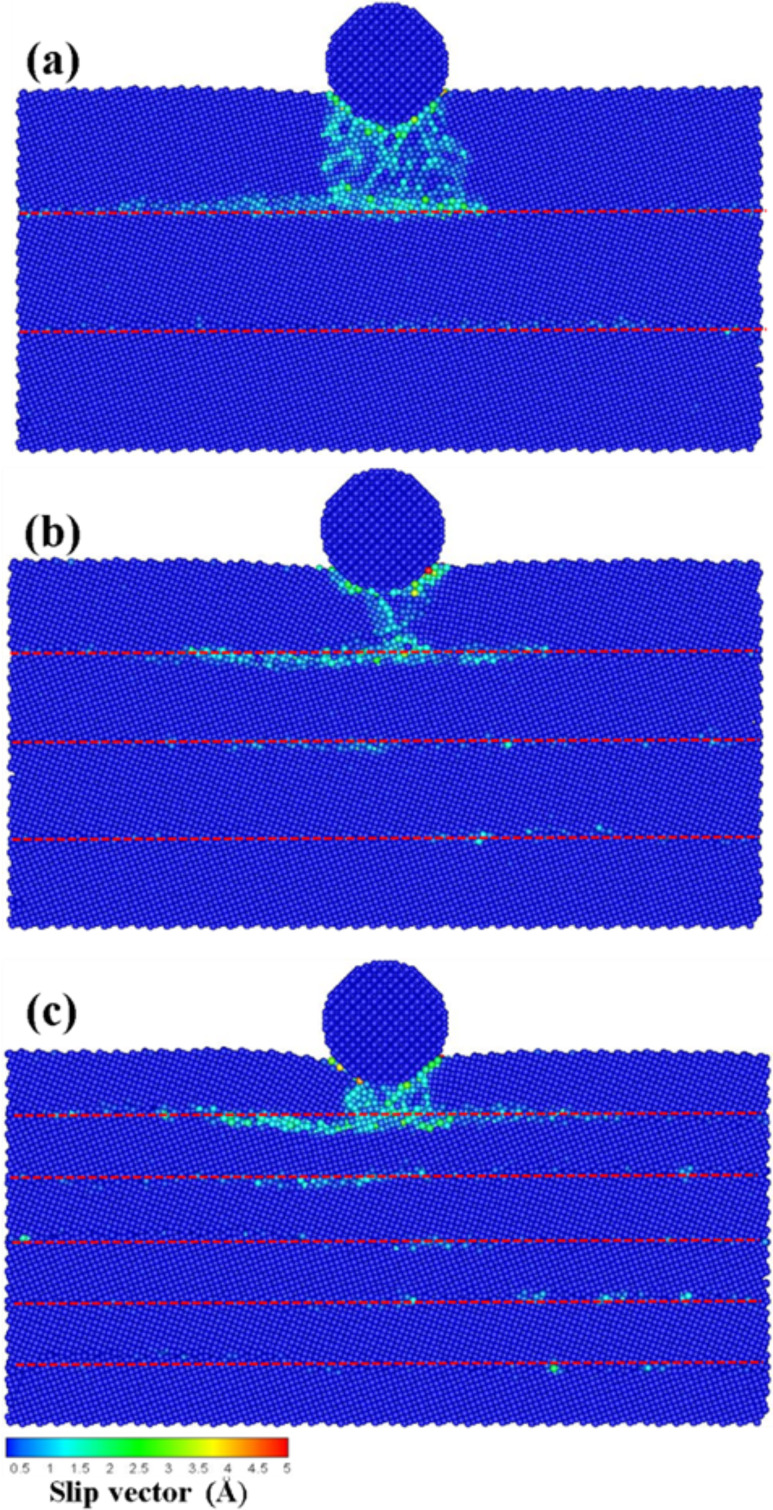
The slip vector diagrams of the transverse grain boundary with the (a) 3 layers, (b) 4 layers, and (c) 6 layers for an indentation of 1 nm. The red dashed line corresponds to the grain boundary.

The effect of the presence of multilayers over the force transmission of the atoms under stress, the direction of the atomic flow for the indentation, and the variation of the normal force is discussed in this section. Figure 5a–c shows the slip vector diagrams of the transverse grain boundaries having 3, 4, and 6 layers, respectively, upon an indentation of 1 nm. The space thickness between the layers was 4, 3, and 2 nm for the 3-, 4-, and 6-layer materials, respectively. As shown in Figure 5, the slip areas were exclusively accumulated on the top of the first layer since the indenter force, which acts over the atoms, was blocked by the harder grain boundary.

**Figure 6:**
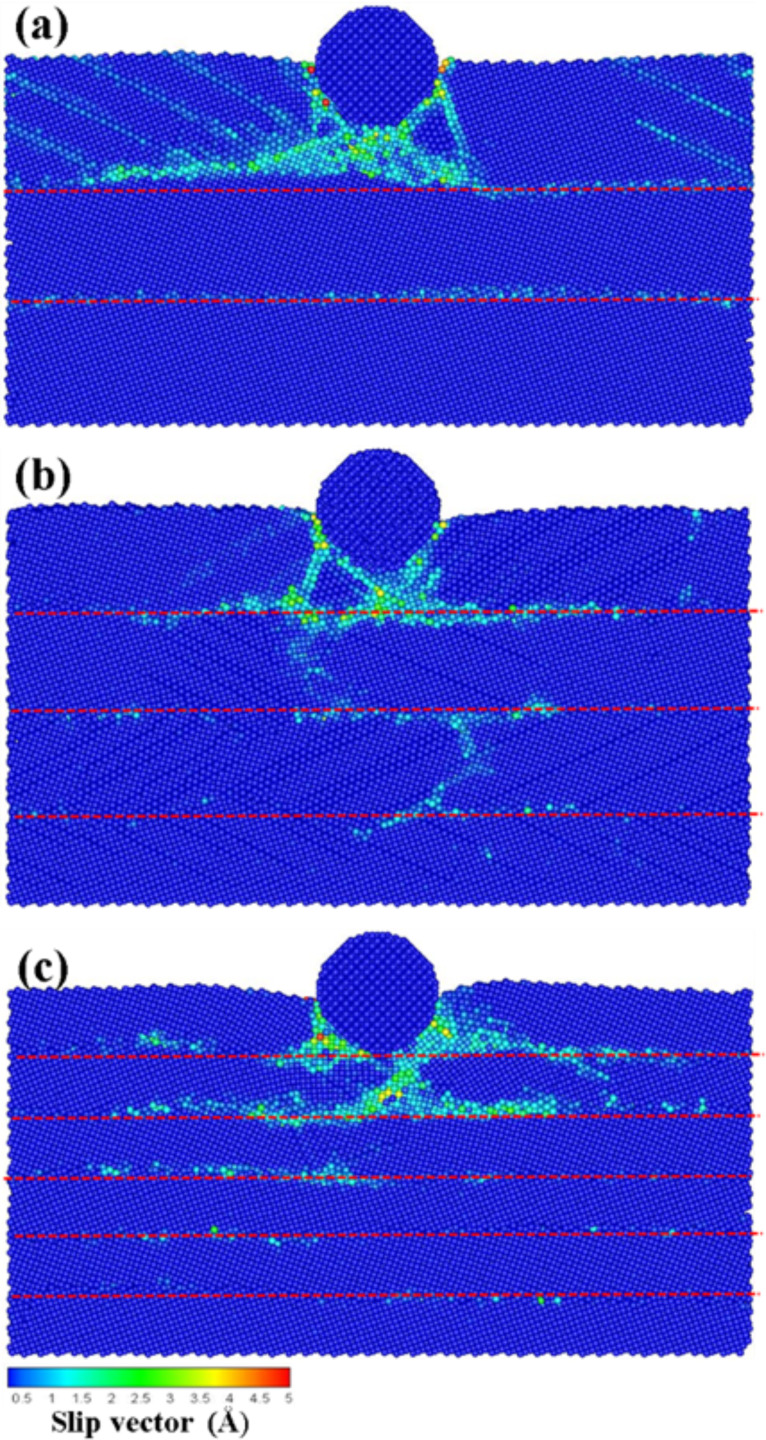
The slip vector diagrams of the transverse grain boundary with the (a) 3 layers, (b) 4 layers, and (c) 6 layers for an indentation of 2 nm. The red dashed line corresponds to the grain boundary.

However, in the case of the 2 nm indentation shown in Figure 6a, the grain boundary resisted the downward movement of the atoms, thereby leading to accumulation of the dislocation on the grain boundary. However, in the case of the 4-layer material (Figure 6b), the atoms of the upper layer moved to the lower layer structure as a result of the depth of the pressure close to the grain boundary, thus leading to the onset of a dislocation. Once the indenter is deep within the grain boundary, the grain boundary breaks as a result of the compression force, thereby preventing the grain boundary from blocking the movement of the dislocation (Figure 6c). Therefore, the atoms of the upper layer passed through the grain boundary and compressed the low layer structure. Moreover, the lower layer structure compressed the dense area, which results in the onset of slip bands.

**Figure 7:**
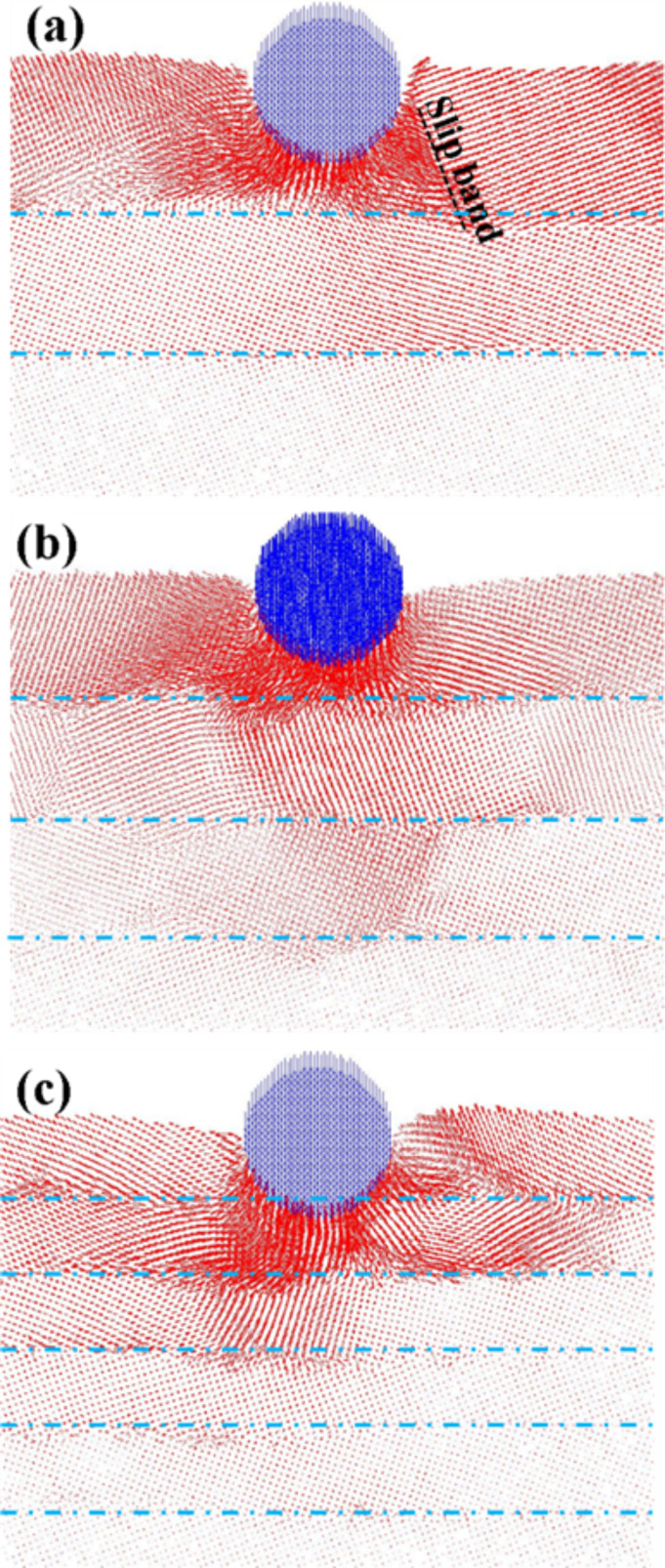
The atomic flow diagrams of the transverse grain boundary with the (a) 3 layers, (b) 4 layers, and (c) 6 layers for the indentation of 2 nm. The blue dashed line corresponds to the grain boundary. The obvious boundary phenomena are marked.

Figure 7a–c presents the atomic flow diagrams of the transverse grain boundaries containing 3, 4, and 6 layers, respectively, upon an indentation of 2 nm. In the case of the grain boundary containing 3 layers (Figure 7a), the atoms of the upper layer moved to the left and right since they were blocked by the grain boundary, thereby leading to the onset of slips bands. Figure 7b,c shows the 4- and 6-layer transverse grain boundaries, respectively. In these cases, the atoms of the upper layer started to move to the lower layer since the grain boundary was compressed by the atoms of the first layer. This behavior led to the onset of a dislocation in the lower layer. This dislocation was more significant in the case of the structure containing 6 layers. This indicates that the force resistance of the grain boundary gradually decreases with the depth of the indentation when it is close to the grain boundary. The slip plane and the dislocation of the atoms of the lower layer were more significant at short distances between the indenter and the grain boundary.

**Figure 8:**
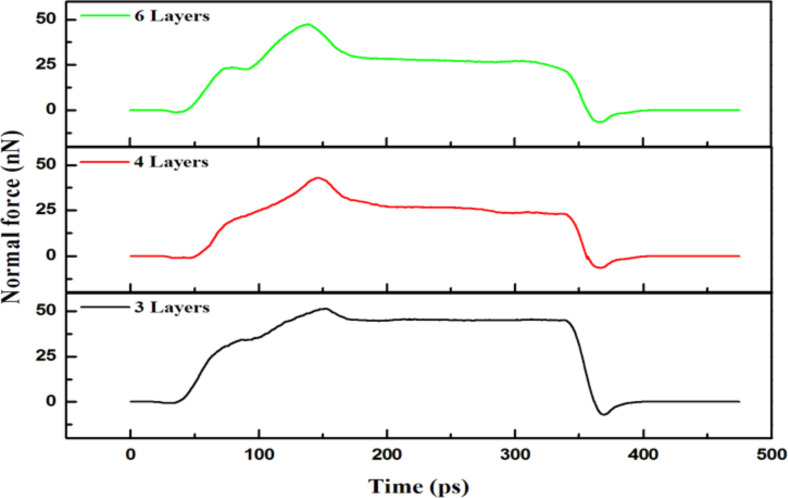
The normal force versus time for the transverse grain boundary with 3, 4 and 6 layers for an indentation of 2 nm.

Figure 8 shows the normal forces as a function of time for the transverse grain boundary containing a varying number of layers upon an indentation of 2 nm. The largest value (≈51.45 nN) appeared in the structure containing 3 layers since the partial pressure from the indenter was distributed along the grain boundary as a result of the blocking effect of the grain boundary for the structures having 4 and 6 layers. However, in the case of the 3-layer structure, the atoms compressed by the indenter were blocked by the harder grain boundary as they moved downward, thus avoiding a smooth transmission of the force and favoring its accumulation on the grain boundary.

### Indentation for the vertical grain boundary

**Figure 9:**
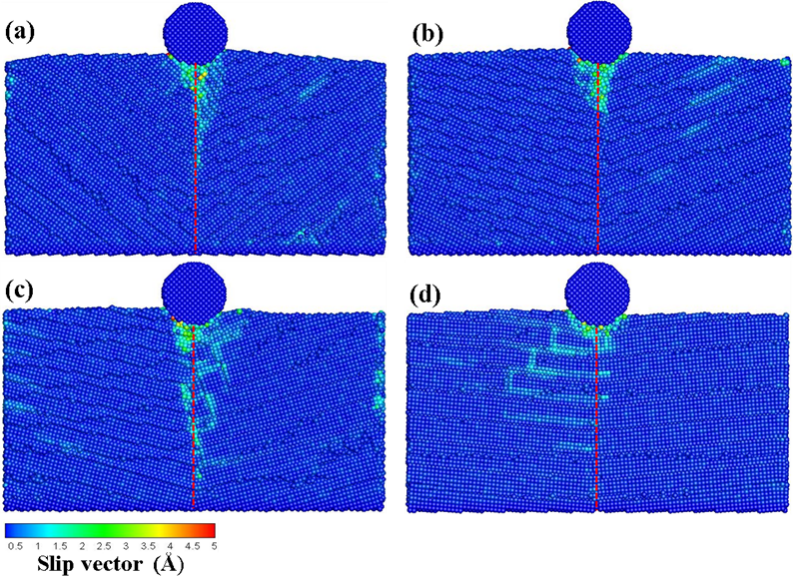
The slip vector diagrams of the vertical grain boundary with the different angles of (a) θ = 10°, (b) θ = 20°, (c) θ = 30°, and (d) θ = 40° for an indentation of 1 nm. The red dashed line corresponds to the grain boundary.

The deformation behavior and the mechanical properties of the crystal layers at the different angles upon pressure from the indenter at the top of the grain boundary are discussed in this section. Figure 9 presents the slip vector diagrams of the vertical grain boundaries at the different angles (θ = 10–40°) upon an indentation of 1 nm. As shown in Figure 9, when the indentation was located at the top of the grain boundary, the atoms slipped along the grain boundary, thereby appearing as intergranular fractures as a result of the faster decay of the strength of the grain boundary as compared to that of the grain structure. The best slip effect of the grain boundary was found for the boundary with a 30° angle. However, the slip area concentrated around the indenter since the grain boundary structure with a 40° angle was similar to that of single crystal copper.

**Figure 10:**
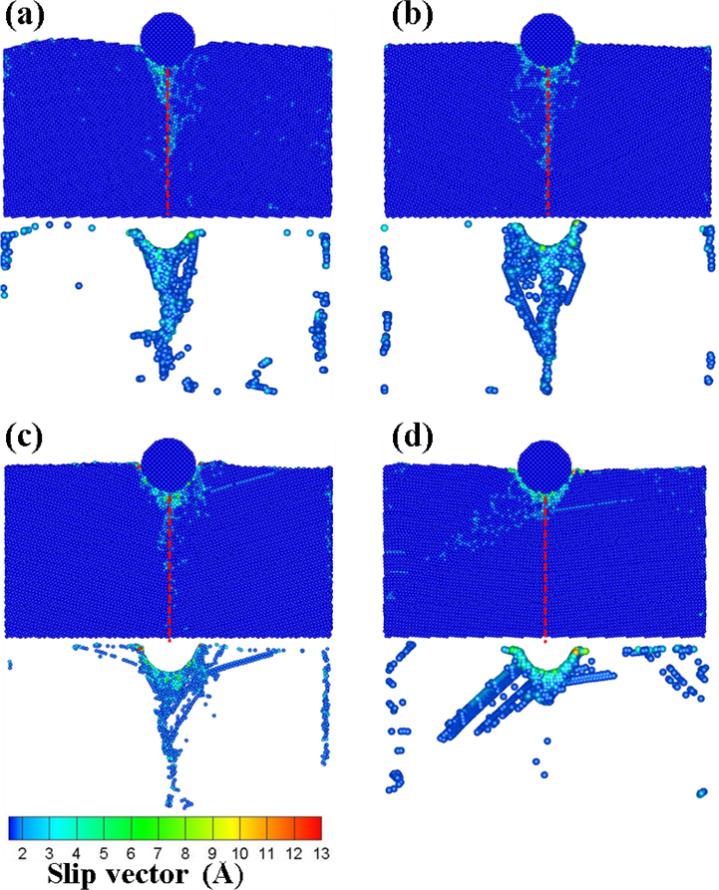
The slip vector diagrams of the vertical grain boundary with the different angles of (a) θ = 10°, (b) θ = 20°, (c) θ = 30°, and (d) θ = 40° for an indentation of 2 nm. The red dashed line corresponds to the grain boundary.

Upon an indentation of 2 nm (Figure 10), the slip area of the grain boundary with a 10° angle is concentrated on the grain boundary, as shown in Figure 10a. In the case of the grain boundaries with 20° and 30° angles (Figure 10b,c, respectively), the dislocation appeared around the location of the indenter and extended to the lower grain boundary. Since the grain boundary structure with a 40° angle (Figure 10d) was similar to the general structure of single-crystal Cu, the atoms did not move along the grain boundary although a large number of dislocation pile-ups appeared around the indenter, thus leading to a new slip plane. This result was in agreement with previous studies showing that the main deformation take place nearly at the grain zones in direct contact with the indenter for a grain size of 8 nm [10]. The authors found that the dislocations piled up at the grain boundary such that the grain boundary supports the strong barriers at large grain sizes [10].

**Figure 11:**
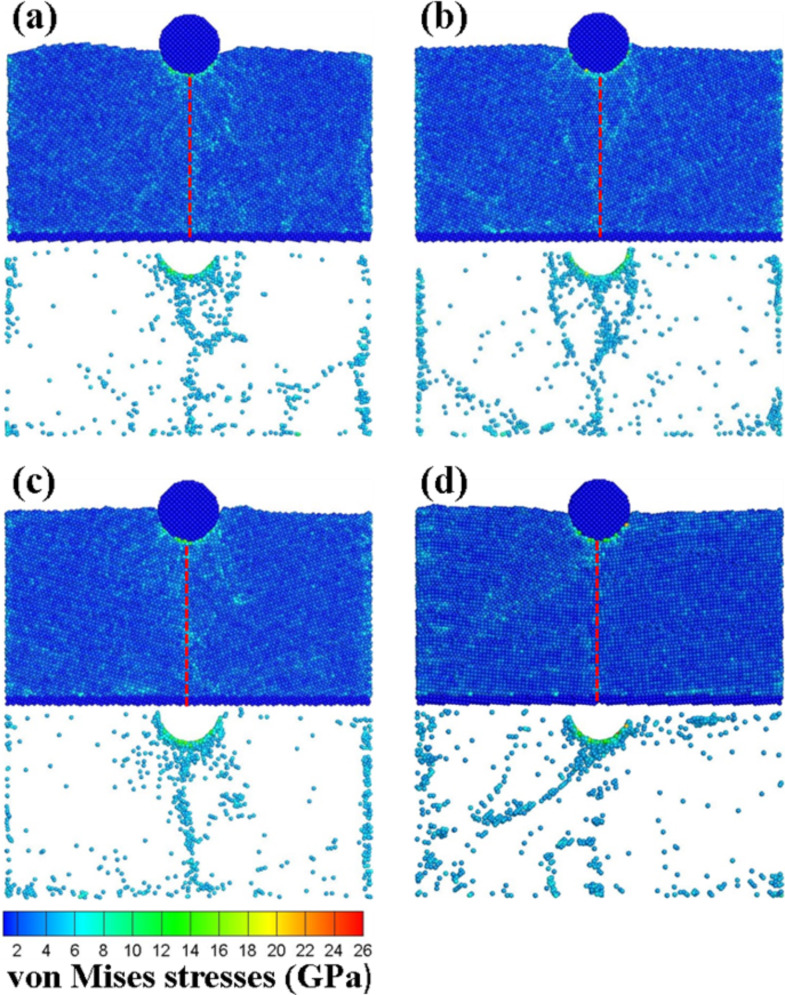
The von Mises stress diagrams of the vertical grain boundary with the different angles of (a) θ = 10°, (b) θ = 20°, (c) θ = 30°, and (d) θ = 40° for an indentation of 2 nm. The red dashed line corresponds to the grain boundary.

Figure 11 shows the von Mises stress diagrams of the vertical grain boundary at different angles (θ = 10–40°) upon an indentation of 2 nm. As shown in Figure 11, the stresses increased with the pressure of the indenter. Since the surface of the substrate was impacted by the pressure of the indenter, the equivalent stresses of the atoms around the indenter were higher as compared to the other zones. Moreover, since the atoms of the substrate were compressed by the indenter, a localized dislocation slip appeared as a result, thus leading to the concentration of the stress. When comparing the results of Figure 10 and Figure 11, it was found that the equivalent stress in the area of the slip bands was relatively large.

**Figure 12:**
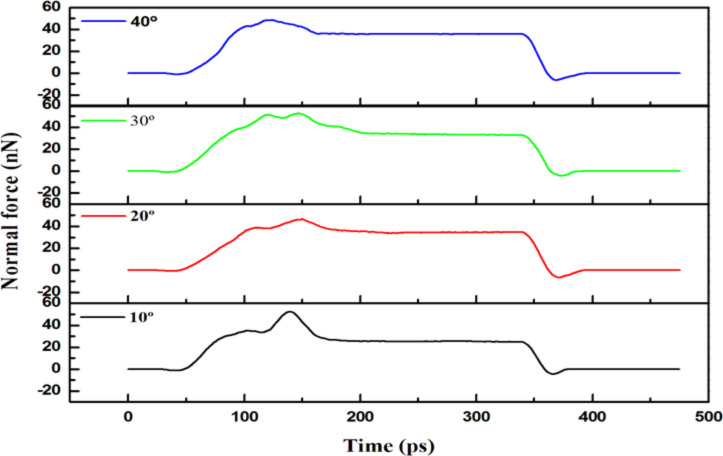
The normal force versus time for the vertical grain boundary with different angles of θ = 10–40° for an indentation of 2 nm.

Figure 12 shows the normal forces as a function of time for the vertical grain boundary at different angles (θ = 10–40°) upon an indentation of 2 nm. Since the force was transmitted along the grain boundary, the destruction of the grain structure was not obvious. Therefore, the indention force should be increased relatively as a result. As shown in Figure 12, the largest value (≈56.23 nN) was obtained in the case of a grain boundary with a 30° angle.

### Scratch for the vertical grain boundary

**Figure 13:**
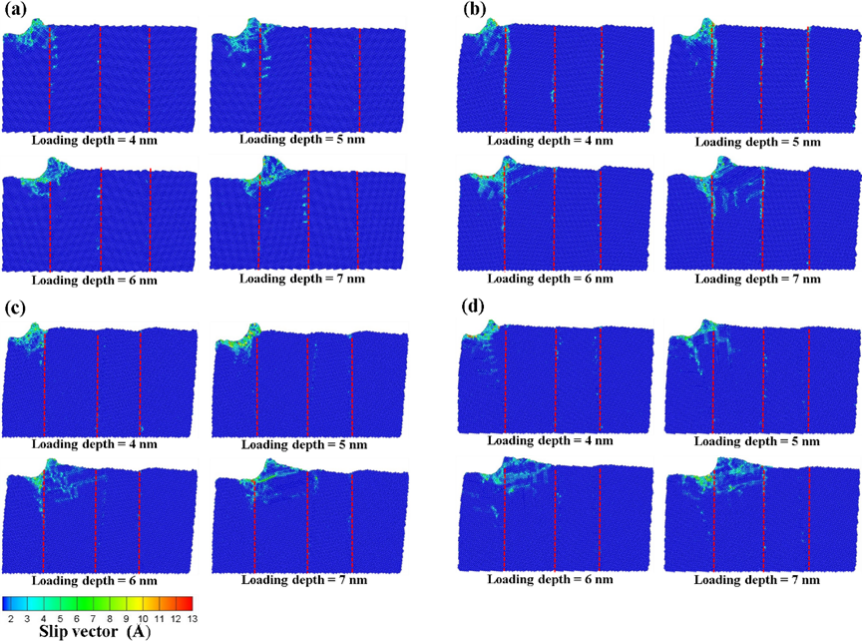
The slip vector diagrams of the vertical grain boundary with the different angles of (a) θ = 10°, (b) θ = 20°, (c) θ = 30°, and (d) θ = 40° for scratchs of 4 to 7 nm.

In this section, we report results where the initial location of the indenter was set at a distance of 1 nm on the left side of the substrate as shown in Figure 1c, and the scratch analysis of the vertical grain boundary was analyzed at different angles (θ = 10–40°) for an indenter speed 100 m/s. Figure 13a–d presents the slip vector diagrams of the vertical grain boundary at different angles for scratch depths ranging from 4 to 7 nm, respectively. As shown in Figure 13a, effects other than slip and pile up (e.g., destruction of the atoms of the grain boundary as a result of the compression) around the indenter were not obvious. The deformation of the grain boundary was clearly appreciated for the 5 nm scratch. Since the atoms behind the grain boundary were destroyed by the scratch, the slip and the pile up increased gradually. The accumulation of atoms was more obvious at higher processing distances. The destruction of the grain boundary restricted the resistance of the force transmission. Moreover, shear bands were found under the zone of accumulation of atoms.

As shown by the slip vector diagrams of the vertical grain boundary with a 20° angle (Figure 13b), the slip was found to be concentrated on the left side of the grain boundary for the 5 nm scratch. No clear dislocation through the grain boundary or new dislocation appeared inside the structure. This behavior can be explained on the basis of insufficient accumulated energy for the dislocation of the grain boundary to penetrate the grain boundary, thereby resulting in the dislocation pile up exclusively appearing on the grain boundary. As the processing distance was increased, the deformation of the substrate atoms still localized on the grain boundary and the zones nearby the indenter. However, the atoms around the grain boundary slipped almost exclusively downward along the direction of the grain boundary. In the case of the 7 nm scratch, since the force crossed the grain boundary, a new dislocation appeared behind the grain boundary of the first layer and extended to the second layer. This therefore led to transgranular fractures [21].

Figure 13c presents the slip vector diagrams of the vertical grain boundary with a 30° angle. In the case of the 5 nm scratch, it can be seen from Figure 13c that the destruction only appeared in the zone under the indenter. However, since the destruction was blocked by the grain boundary, the structure behind the grain boundary was not affected except for the atoms that accumulated as a result of the scratch. On the other hand, in the case of the 6 nm scratch, the indenter was located at the top of the grain boundary. Since the grain boundary was destroyed by the scratch force, the force passed through the grain boundary and the slip plane appeared behind the grain boundary. In the case of the 40° angle scratch (Figure 13d), the effect of the force resistance of the grain boundary was not satisfactory. In the case of the 5 nm scratch, the back of the grain boundary was destroyed by the dislocation. Moreover, the slip areas increased with the distance to the scratch because the structure properties were similar to that of a normal single-crystal Cu.

**Figure 14:**
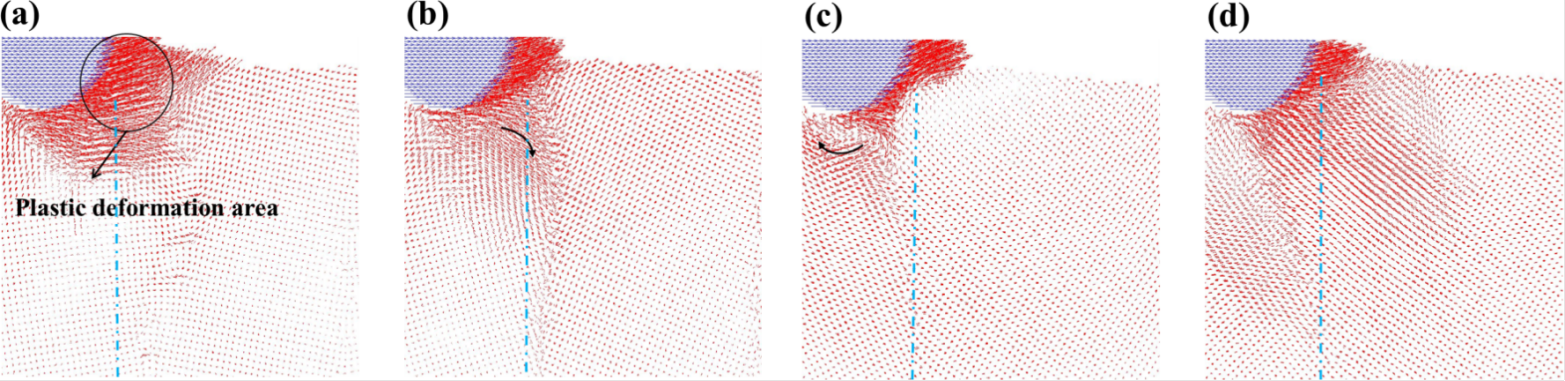
The atomic flow diagrams of the vertical grain boundary with the different angles of (a) θ = 10°, (b) θ = 20°, (c) θ = 30°, and (d) θ = 40° for a scratch of 5 nm. The blue dashed line corresponds to the grain boundary. The curved arrow indicates the direction of the atomic flow.

With the aim to further understand the situation of the atomic flow inside the structure, the atomic flow diagram at the different angles for the 5 nm scratch was used, as shown in Figure 14. In the case of the 10° angle scratch (Figure 14a), the atoms of the grain boundary compressed by the indenter moved to the left. The atoms continued to be compressed on the left side without resistance of the grain boundary, thereby leading to plastic deformation. This result was in agreement with the study of Chu et al*.* [14]. On the other hand, the atoms under stress were accumulated by the scratch owing to the strength of the structure of the grain boundary with a 20° angle (Figure 14b). Moreover, the atoms under the indenter were blocked by the grain boundary and they moved along the grain boundary, thereby leading to intergranular fractures. When comparing the atomic flow of the 20° and 30° angle materials, it can be seen that the atoms affected by the push of the indenter flowed into the grain boundary. Although the atomic flow for the material with a 30° angle (Figure 14c) was blocked by the grain boundary, there were no intergranular fractures. Instead, a pile up phenomena appeared ahead of the indenter owing to the lifting up of the atoms located in front of the indenter. However, the atoms under the indenter slipped backward owing to the grain boundary blocking effect. In the case of the material with a 40° angle (Figure 14d), it was found that the atoms under the force all moved along the processing direction. Thus, this material did not significantly block the transmission of the force.

**Figure 15:**
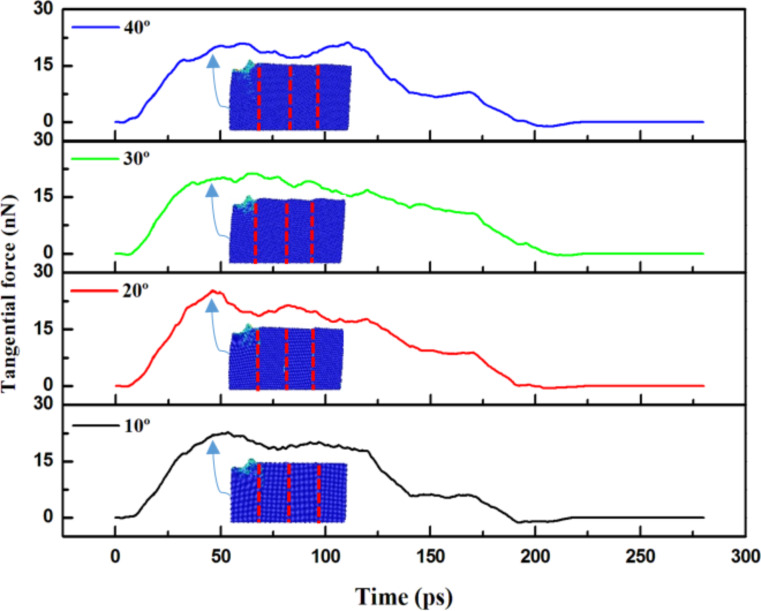
The tangential force versus time for the vertical grain boundary at different angles θ = 10–40° for a scratch of 5 nm. The inserts are the slip vector diagrams of 5 nm for 10°, 20°, 30° and 40° angles corresponding to Figure 13.

Finally, Figure 15 shows the tangential forces as a function of time for the 5 nm scratch. The arrows and inserts show the positions of scratch, which respond to the slip vector results of 5 nm for 10°, 20°, 30° and 40° angles as shown in Figure 13. As shown in Figure 15, the force was higher as the scratch approached the grain boundary owing to the blocking effect exerted by the grain boundary. The force started to gradually decrease after it crossed the grain boundary. As shown in Figure 15, the highest force values (≈25.14 nN) appeared in the grain boundary with a 20° angle.

**Figure 16:**
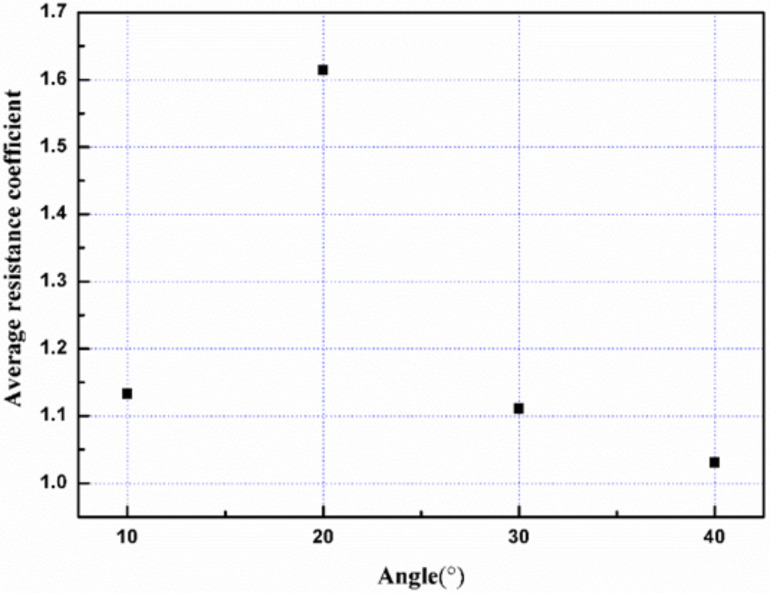
The average resistance coefficient versus the different angles for a scratch of 5 nm.

The average resistance coefficient (δ) shown in Figure 16 was studied to compare the resistance of the scratch at different angles. δ is defined as the tangential force (*f*_y_) divided by normal force (*f*_z_). As shown in Figure 16, the highest value of the average friction coefficient was observed for the grain boundary with a 20° angle. Therefore, in the case of vertical grain boundaries at different angles, it can be deduced that the structure strength of the grain boundary with a 20° angle is stronger as compared to other structures.

## Conclusion

The mechanical and grain boundary characteristics of Cu films under nanoindentation and scratch conditions were studied using MD simulations. The conclusions of the analysis and the deformation mechanism of the grain boundary are as follows.

In the case of the nanoindentation of the transverse grain boundaries, since the grain boundary has a higher strength, the transmission of the force will be limited when passing through the grain boundaries. The existence of the grain boundary prevents the movement of the dislocation, while also leading to plastic deformation of the material but with difficulty.

In the case of the nanoindentation of the transverse grain boundaries at different angles, the structure of the grain boundary with a 20° angle was stronger, thereby limiting the force to the slip direction of the upper dislocation. Therefore, the deformation of the structure was largely dispersed in the horizontal direction, which makes the lower structure difficult to disrupt.

From the simulation results of the grain boundary with multilayers, it was found that when the material is destroyed, a deformation behavior always appeared near the indenter along with a squeeze phenomenon. The deformation passed through the grain boundary to the underlying layers when the squeeze force was large enough.

In the case of the nanoindentation of the vertical grain boundaries at different angles, when the stress point was located on the grain boundary, intergranular fractures were produced along the grain boundary since the decay of the structural strength of the grain boundary was faster than that of the nearby grain boundary. Intergranular fractures were more evident in the case of the grain boundary 20°.

According to the nanoindentation and nanoscratch simulation results, the existence of the grain boundary enhanced the mechanical properties of the material while also increasing the resistance to the movement produced by the internal dislocation. This behavior led to the accumulation of the dislocation on the grain boundaries.
